# Chronic Neurology in COVID-19 Era: Clinical Considerations and Recommendations From the REPROGRAM Consortium

**DOI:** 10.3389/fneur.2020.00664

**Published:** 2020-06-24

**Authors:** Sonu Bhaskar, Sian Bradley, Simon Israeli-Korn, Bindu Menon, Vijay Kumar Chattu, Pravin Thomas, Jasvinder Chawla, Rajeev Kumar, Paolo Prandi, Daniel Ray, Sailaja Golla, Nirmal Surya, Harvey Yang, Sandra Martinez, Mihriban Heval Ozgen, John Codrington, Eva María Jiménez González, Mandana Toosi, Nithya Hariya Mohan, Koravangattu Valsraj Menon, Abderrahmane Chahidi, Susana Mederer Hengstl

**Affiliations:** ^1^Pandemic Health System REsilience PROGRAM (REPROGRAM) Consortium, Chronic Neurology REPROGRAM Sub-committee^†^; ^2^Department of Neurology and Neurophysiology, Liverpool Hospital, Sydney, NSW, Australia; ^3^Neurovascular Imaging Laboratory & NSW Brain Clot Bank, Ingham Institute for Applied Medical Research and South West Sydney Clinical School, The University of New South Wales, UNSW Medicine, Sydney, NSW, Australia; ^4^The University of New South Wales, UNSW Medicine, Sydney, NSW, Australia; ^5^Department of Neurology, Sheba Medical Center, Tel Hashomer, Ramat Gan and Sackler School of Medicine, Movement Disorders Institute, Tel Aviv University, Tel Aviv-Yafo, Israel; ^6^Department of Neurology, Apollo Hospitals, Nellore, India; ^7^Department of Medicine, St. Michael's Hospital, University of Toronto, Toronto, ON, Canada; ^8^Department of Neurology, University Hospitals NHS Foundation Trust, Birmingham, United Kingdom; ^9^Department of Neurology, Loyola University Medical Center & Hines VA Hospital, Chicago, IL, United States; ^10^Department of Psychiatry, Hamad Medical Center, Qatar & Australian National University, Canberra, ACT, Australia; ^11^Department of Neurology, University of Eastern Piedmont Amedeo Avogadro, Novara, Italy; ^12^Farr Institute of Health Informatics, University College London (UCL) & NHS Foundation Trust, Birmingham, United Kingdom; ^13^Texas Institute for Neurological Disorders, Dallas, TX, United States; ^14^Department of Neurology, Bombay Hospital & Medical Research Centre, and Epilepsy Foundation India, Mumbai, India; ^15^Department of Neurology, Academic Hospital Paramaribo & Anton de Kom Universiteit van Suriname Faculteit der Medische Wetenschappen, Paramaribo, Suriname; ^16^Department of Neurology, Hospital da Restauração, Recife, Brazil; ^17^Department of Psychiatry, Parnassia Psychiatric Institute, The Hague, Netherlands; ^18^Curium-Leiden University Medical Centre, Oegstgeest, Netherlands; ^19^Department of Laboratory Medicine, Academic Hospital Paramaribo and Anton de Kom Universiteit van Suriname Faculteit der Medische Wetenschappen, Paramaribo, Suriname; ^20^Department of Forensic Psychology, Forensic Psychology and Forensic Sciences Institute, Ministry of Justice, Granada, Spain; ^21^LodeStone Center for Behavioral Health and Eastern Illinois University, Chicago, IL, United States; ^22^Chengalpattu Medical College and Hospital, Chengalpattu, India; ^23^Department of Psychiatry, South London and Maudsley NHS Foundation Trust, Kings Health Partners, London, United Kingdom; ^24^ED 268, DR 178, Sorbonne Nouvelle University, Paris, France; ^25^Moroccan Society of Neurophysiology, Marrakech, Morocco; ^26^Morocco and Basic and Clinical Neurosciences Research Laboratory, University Medical School of Marrakech, Marrakech, Morocco; ^27^Department of Neurology, Complejo Hospitalario de Pontevedra, Pontevedra, Spain

**Keywords:** coronavirus disease 2019 (COVID-19), chronic neurological disease, healthcare services, guidelines, neurodegenerative disorders, protocols, pandemics, recommendations

## Abstract

With the rapid pace and scale of the emerging coronavirus 2019 (COVID-19) pandemic, a growing body of evidence has shown a strong association of COVID-19 with pre- and post- neurological complications. This has necessitated the need to incorporate targeted neurological care for this subgroup of patients which warrants further reorganization of services, healthcare workforce, and ongoing management of chronic neurological cases. The social distancing and the shutdown imposed by several nations in the midst of COVID-19 have severely impacted the ongoing care, access and support of patients with chronic neurological conditions such as Multiple Sclerosis, Epilepsy, Neuromuscular Disorders, Migraine, Dementia, and Parkinson disease. There is a pressing need for governing bodies including national and international professional associations, health ministries and health institutions to harmonize policies, guidelines, and recommendations relating to the management of chronic neurological conditions. These harmonized guidelines should ensure patient continuity across the spectrum of hospital and community care including the well-being, safety, and mental health of the patients, their care partners and the health professionals involved. This article provides an in-depth analysis of the impact of COVID-19 on chronic neurological conditions and specific recommendations to minimize the potential harm to those at high risk.

## Introduction

**Figure 1:**
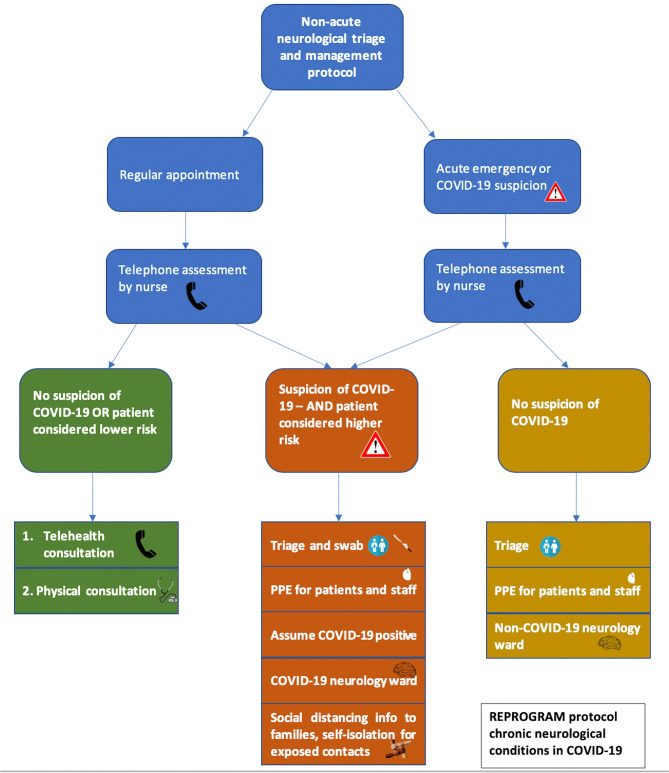
Proposed flow chart outlining the triage and management decisions for patients with chronic neurological conditions in the COVID-19. This flow chart applies to chronic patients who need to make a regular appointment, such as for scheduled check-ups or prescription refills, or for acute emergency presentations due to possible COVID-19 cases or acute neurological symptoms. In both situations, an initial telephone conversation should screen patients to assess the possibility of a COVID-19 infection. This will involve asking about fever, cough, sore throat, fatigue, shortness of breath, anosmia, and potential COVID-19 contact. For those patients without COVID-19 symptoms or who are at low risk and don't require presentation to hospital, telehealth consultation with their neurologist or primary physician can be undertaken. Where this is not possible, physical consultation may be required. If there is suspicion that the patient may have COVID-19 and they are considered at higher risk to their health, they should present to the hospital and receive point-of-entry triage and swabbing for COVID-19 infection. PPE should be worn by both staff and patients. Patients should be assumed to be COVID-19 positive until proven otherwise and be taken to a designated COVID-19 neurology ward. Close relatives and attendees of the patient should also practice social isolation whilst awaiting test results. For patients who require acute neurological care and are not suspected of having a COVID-19 infection, they should receive point-of-entry triage, PPE should be supplied to patients and staff and they should be admitted to a non-COVID-19 neurology ward if necessary. Triage assessment will involve stratification by mild, moderate, severe, and critical risk, according to Table 1.

Coronavirus 2019 (COVID-19), officially severe acute respiratory syndrome (SARS) associated coronavirus (SARS-CoV2) (1), was declared by the World Health Organization (WHO) to have reached pandemic status on the 11th March 2020 (2). The global reach of the disease continues to promote fear and panic amongst members of the public and healthcare workers. In the COVID-19 pandemic, chronic neurological care is increasingly under stress due to ongoing reorganization and rationing of services to meet the demands of frontline COVID-19 cases. Patients with chronic neurological diseases are forced to balance their pre-existing conditions with this rapidly evolving threat of COVID-19 (3, 4). Specific considerations for clinical management of these patients and of health services are warranted in the background of huge social and economic costs associated with long-term morbidity. This article pursues to discuss the ongoing approaches to neurological patient management in the COVID-19 era and provides comprehensive recommendations for specific chronic neurological conditions. Patients with chronic neurological diseases such as Multiple Sclerosis, Epilepsy, Neuromuscular Disorders, Migraine, Dementia, and Parkinson disease would benefit from a targeted strategy to minimize harm and prevent long-term associated costs to society and to the economy. Mental health implications of COVID-19 on chronic neurological patients and healthcare workers are also discussed. A triage and management protocol for chronic neurological patients presenting to the emergency in the COVID-19 period is also proposed (Figure 1).

## Current Approaches in Chronic Neurological Patients During COVID-19

COVID-19 positive patients could be classified into three categories depending upon the presenting neurological symptoms: neurological manifestations in patients with underlying disease [headache, dizziness, impaired consciousness (5), ataxia, seizures or epileptic manifestations (6, 7), and stroke (5)]; neuro-peripheral origin associated neurological expressions (hypo-ageusia, hyposmia, neuralgia) and symptoms of skeletal muscle damage (5, 8, 9). The prevalence rates of hypogeusia and hyposmia in COVID-19 patients are variable in the literature. The prevalence of hyposmia and hypogeusia was 5.1 and 5.6% in a study on 214 hospitalized patients with COVID-19 from Wuhan (China), respectively (5). In a multi-center European study on 417 mild-to-moderate COVID-19 patients, olfactory, and gustatory dysfunctions were reported by 85.6 and 88% of patients, respectively (10). Hyposmia or anosmia was present in ~78% of patients without nasal obstruction or rhinorrhoea (10). A Korean study reported a prevalence of acute anosmia or ageusia in 15.3 and 15.7% in the early stage of COVID-19 and in patients with asymptomatic-to-mild disease severity, respectively (11). Chemosensory dysfunction was present in ~19.4% of patients in Italy (12). With emerging evidence concerning anosmia presenting as an early symptom of COVID-19, dedicated testing for anosmia could be useful in early detection of COVID-19 infection (9).

Several countries have imposed strict social distancing measures. In the United Kingdom (UK), strict social distancing is recommended for those with chronic neurological conditions, such as Multiple Sclerosis (MS), Motor Neuron Disease (MND), Myasthenia Gravis (MG), inflammatory myopathies, autoimmune neuropathies, epilepsy, Parkinson's Disease (PD), Alzheimer's disease, and immunosuppressed individuals (13). Social distancing or quarantine could have further detrimental effects both mentally and physically on patients who require physical therapy, mobilization, and assistance as these resources become increasingly difficult to access or become less available.

A number of approaches to the management of patients with chronic neurological conditions during the time of COVID-19 have been explored worldwide to maintain patient continuity. Measures including remote triaging, reliance on telemedicine for outpatient consultations, separation of COVID and non-COVID patients in emergency departments (ED) and working across subspecialties are being applied (14). Such patients and those with vascular comorbidities need to be supported so that issues such as discontinuing previous medications out of fear, canceled outpatient appointments, and general anxiety can be managed. The Association of British Neurologists has released an outline of risks posed to chronic neurological patients generally, as well as risk stratification of particular diseases (15). Those presented with higher risks include those with high doses of immunotherapy, multiple immunotherapies, active disease, swallowing or respiratory muscle weakness, and comorbidities (15). The American Academy of Neurology (AAN) has also issued guidelines on telemedicine for management of chronic neurological patients (16). The American Headache Society recommends the use of telemedicine where possible for COVID-19 symptoms screening and recommends the need for triage for the presentation to clinics (17). In the advent of an emergency during the COVID-19 era, patients with chronic neurological conditions should be separated from non-neurological patients, due to the greater risk posed to their health (18). We will now critically examine the impact of COVID-19 on various neurological conditions and provide strategies and recommendations to improve patient management and reduce negative impact during the current pandemic.

## Multiple Sclerosis

Multiple Sclerosis is a neuroinflammatory disorder, which confers risk to those affected due to the prevalence of immunotherapy, as well as potential swallowing and breathing difficulties. Media reports have shown patients with MS to be anxious given their immunocompromised state and the reduced availability of services in the COVID-19 crisis (19). This is a worldwide concern, as, amongst young people, MS is the most common neurological cause of disability, with the highest prevalence and incidence rates being in Europe with over 750,000 affected individuals (20, 21). To alleviate some concern, several MS associations have released guidelines on disease-modifying therapy (DMTs) (15, 22). Patients may be tempted to cease their immunotherapy due to fears of infection risk, however, the general consensus amongst physicians and associations is that changes to medication should not be made without consulting their neurologist. In the case that patients become infected with COVID-19, it might become necessary to cease immunotherapy depending on the severity of the infection. Certain DMTs may be considered immunomodulators, with little to some immunosuppression abilities, whilst others are considered definitive immunosuppressants (23, 24). It is unknown what risk immunosuppression gives in relation to COVID-19, however, it remains prudent for those with MS to consult their physicians and practice social-distancing and good hygiene practices.

The move to telemedicine for neurological patients can be helpful in the ongoing management of MS patients. The Northwestern University Multiple Sclerosis Clinic has demonstrated the ability to rely on telemedicine for such patients, with exceptions including necessary transfusions and potential relapse investigation (25). A further area for consideration is in the cognitive health of COVID-19 positive patients, and whether the COVID-19 induced infection will accelerate the severity of the MS and/or hence the associated increased rate of brain atrophy. Age-matched and severity-matched comparisons of COVID-19 positive and COVID-19 negative chronic neurological patients could help determine whether there is a putative long-term impact.

## Neuromuscular Diseases

Patients with neuromuscular diseases are at heightened risk of COVID-19 infection. No data currently exist on how COVID-19 affects people with neuromuscular disorders including MND, MG, autoimmune or inflammatory neuropathies, or inflammatory myopathies, etc. There are no clear guidelines as to how this will impact patients taking immunosuppressive therapies. We need to assume that patients on immunosuppressive therapies and/or with bulbar/respiratory muscle weakness such as MG or Lambert Eaton myasthenic syndrome, are at higher risk of contracting the infection or experiencing severe manifestations of COVID-19 (26). Patients with MND often suffer severe disability involving bulbar or respiratory muscle weakness and are hence considered at a higher risk, particularly due to the threat of pneumonia development in the COVID-19 scenario (27).

Furthermore, some patients may already require interventions such as non-invasive ventilation (NIV) or bilevel positive airway pressure (biPAP), and it is essential that any personal breathing equipment is being regularly cleaned and maintained. The American Academy of Sleep Medicine recommends that the use of continuous positive airway pressure (CPAP) and biPAP in a home setting should only be undertaken by COVID-19 patients who already used PAP at home, and it should be done so in an isolated room (28). Neuromuscular patients who are dependent on biPAP may also require use in hospitalization. There is a lack of international consensus on guidelines despite the availability of a considerable number of recommendations.

The International MG/COVID Working Group have provided guidance on patients with MG and Lambert Eaton myasthenic syndrome (LEMS) and the use of therapies during the COVID-19 pandemic (26). It is anticipated that some patients with neuromuscular diseases who require maintenance infusions would require hospital or infusion center visits. From a health system organizational perspective, health care providers need to consider providing information on protocols concerning access to these facilities and precautions that must be adhered by those available in them. Alternative options such as home infusion could be considered. However, this may require additional training for self-administration that could be provided remotely using tele-neurology. Immunoglobulin infusion and/or plasmapheresis haven't been shown to increase potential COVID-19 infection risk. Routine blood monitoring required with certain medications needs to be streamlined and based on individual needs and regional COVID-19 prevalence. The possibility of corticosteroid dose escalation in MG patients might be considered during COVID-19 times. Temporary suspension of immunosuppressive therapies would be required in patients with comorbid sepsis-associated hospitalization. Pharmacological profile and characteristics, such as longer wash-out periods and rebuilding therapeutically optimal levels on resuming therapy of individual immunosuppressive drugs need to be weighed (26). In essence, the treatment escalation or change decisions need to be individualized based on the relative severity of COVID-19 infection and underlying neuromuscular disorder.

Patients with neuromuscular diseases are advised to adhere to public health measures as invoked by their respective local and national bodies. Patients should be reassured to continue their ongoing treatment and any changes to the treatment regimen and dosage should only be made on the advice and approval of the treating physician. Patients should be educated and advised to follow strict social distancing, regular handwashing, and avoiding non-essential travel (27). Online order facilities to procure groceries/daily provisions and stocking up on non-perishables to reduce grocery trips should be pursued. It is advised that patients have adequate stock of essential items, such as medication and feeding-tube supplies. A concurrent issue is that many MND patients are reliant on carers. Recommendations by the New Zealand MND organization to ensure continuity of care include having a list of medications, dosages and healthcare providers; ensuring strict hygiene of self and environment and utilization of MND support resources in addition to familial and community support (29).

The impact on individual patients with neuromuscular disease relying on novel treatment remains to be seen. MND poses a unique worry due to a lack of curative treatment options and the dependence of a substantial number of patients on clinical trials. There is an indication that some clinical trials have been halted, as well as a lack of new trial or recruitment opportunities. It should, therefore, be recognized that there may be some distress in the MND community at this time and that ongoing support will be required. It is hoped that this will only be a temporary issue, however, the impact on individual patients relying on novel treatment remains to be seen.

## Epilepsy

There are conflicting opinions as to whether patients with epilepsy are considered more vulnerable to the disease than the general population (30, 31). Whilst some epilepsy organizations do not consider this population especially vulnerable, it is important to recognize that patients with epilepsy are widely varied in their presentations and a significant proportion (~80%) of epilepsy patients live in a low or middle-income country (LMIC) (32). Patients with seizures that are triggered by fever and infection may be considered at a relatively higher risk and could consider taking antipyretics if a fever develops. As typical antiepileptic medications do not suppress the immune system, it is imperative that patients do not cease their anti-epileptic drugs (AED) for fear of COVID-19 or due to inability to get scripts (33). In the present circumstances, any patients with new-onset seizures should be tested for COVID-19 as it has been reported that COVID-19 could present as seizures due to encephalopathy, however, patients should be treated as per epilepsy protocol with AED (34). Concern for patients is the unavailability of AEDs and there need to be guidelines from the government to allow pharmacists to refill old prescriptions. This is important as many people living in rural areas may not have smartphone access to digital prescription. The use of telemedicine could be key for follow up, guidance and counseling for people with epilepsy.

If a patient has mobility or cognitive disabilities associated with their epilepsy, they could also be considered vulnerable and should avoid exposure and be provided with support. Given the disproportionate burden of epilepsy in LMIC countries, relatively limited access and provision of specialist care and absence of targeted and available guidelines in their respective languages from the epilepsy organizations and support networks; it is notable that patients with epilepsy may be at a significantly higher disadvantage in these specific regions. In case of expected bed shortages, rationing of non-acute neurological testing including cancellation of elective epilepsy monitoring could be explored. However, individual case-mix and case-by-case approach are preferred as deferring diagnostic tests or monitoring could result in a possible case scenario where a patient might end up having a severe episode of seizure that would require emergency admission. While these measures are taken globally, the Epilepsy Foundation has also provided some guidelines, including when to go to the emergency room, medications and common concerns about COVID-19 and epilepsy (35).

## Parkinson'S Disease and Other Movement Disorders

Patients with Parkinson's Disease (PD), although not at higher risk *per se*, should be considered a vulnerable population due to older age, bulbar symptoms, respiratory dysfunction, frailty and cognitive impairment with respect to measures taken to minimize their potential exposure to COVID-19 as well as to ensure monitoring for compliance (36). Patients with PD are more likely to develop pneumonia, and infections can lead to sudden motor and cognitive changes. Healthcare workers treating COVID-19 patients should, therefore, be equipped to manage PD patients, as well as being prepared for potential cases of delirium (37).

Notably, PD is associated with anosmia often prior to diagnosis (38), and hence this may not be a useful indication of potential COVID-19 infection (39). Nursing and care homes need to ensure that PD patients remain in quarantine. Continuation of Botox® for symptomatic control of dystonia and post-stroke spasticity should be made on a case by case basis. Due to close proximity between the physician and the patient, both masks and eye protection should be used (40). Special considerations also apply for invasive procedures. This should be on a case-by-case basis, but non-urgent elective procedures: percutaneous endoscopic gastrostomy (PEG) for Duodopa, focused ultrasound thalamotomy (FUT), and deep brain stimulation (DBS) should be postponed, if and whenever possible. However, implantable pulse generator (IPG) battery replacements should still be performed because of the risks of the neuroleptic malignant syndrome with sudden battery failure (41).

Lastly, along with the importance of telemedicine in these patients to ensure continuity of care, tele-exercise and tele-physiotherapy are very important in patients with PD as well as other clinical populations with neurological and non-neurological chronic disease. The psychosocial aspects of the impact of COVID-19 infection and of increased social isolation and sudden change to the daily routine, are of particular clinical relevance in Parkinson's disease patients. On the other hand, with adequate psychosocial support, the experience of being isolated at home together with everyone else in the country may, in fact, be somewhat normalizing in the sense that being socially isolated (owing to mobility constraints or stigma/shame relating to their diagnosis/symptoms), no longer results in them experiencing the feeling of missing out.

## Migraine and Severe Headaches

Migraine and severe headaches are one of the most frequent outpatient presentations in neurology clinics. To minimize risks of infection to healthcare workers, patients and the health system, all non-emergent procedures (such as Botox® injections) should be deferred. During the COVID-19 period, the goal is to keep people at home with the appropriate treatment provided via telemedicine unless absolutely essential to bring patients to hospitals. Face to face consultations and follow-up visits should be done remotely via telephone/video call (telemedicine) (42). A recent randomized control trial showed telemedicine was an effective mode of treatment and improves physician productivity and patient satisfaction (43). Treating neurologists and/or headache specialists must maintain and encourage patient-continuity using telemedicine such as through video or telephone visits. Tele-consultation for worsening headaches should include a red-flag checklist for secondary headaches: only those with red/orange/yellow flags should be brought in, prioritized in that order. In resource-constrained settings, where there is a lack of manpower for doctors to deal with headaches, specialist nurses/physician assistants and junior doctors could be assigned to take calls.

Botox® should be deferred for all migraine patients; however, “high-risk” patients, especially those with severe depression and/or at suicide risk, who are likely to come to the emergency in the advent of a severe headache episode could be considered for outpatient Botox® therapy to limit exposure. Should a need for urgent in-person patient consultation be felt by the treating physician, remote screening for COVID-19 symptoms and any previous history of travel or contact with a COVID-19 infected person could be done over the phone. All acute headache presentations must be screened, along with a swab for COVID-19, should there be any suspicion of COVID-19. Concomitant concerns have been raised regarding the use of ACE2-stimulating drugs, such as renin-angiotensin system (RAS) blockers and the non-steroid anti-inflammatory drug (NSAID) ibuprofen, and an increased risk of COVID-19 infection and developing severe fatal COVID-19 viral infection (44, 45). Patients with migraine and severe headaches should not change therapy unless clinically indicated (44, 46). In COVID-19 infected patients, ACE2-stimulating drugs should be switched to another drug, such as specific human immunoglobulin under close clinical supervision, until the infection abates (47). For acute migraines in suspected or confirmed COVID-19 cases, NSAIDs and beta-blockers (owing to bronchospasm complications in COVID-19 associated respiratory flare) should be avoided (48). However, it's important to note that termination of beta-blockers can cause withdrawal symptoms and have been linked to death in specific patients with cardiac conditions such as unstable angina and those who underwent coronary bypass surgeries (49–51).

Triptans should be used with caution in males with hypertension and older age because of the greater risk of stroke with COVID-19 (52, 53). Paracetamol (acetaminophen) with antiemetics are recommended for acute migraine headaches as this should relieve nausea and vomiting (54). Caution should be exercised in the use of steroids for non-COVID-19 patients as these patients would be immunocompromised with their use.

There is a propensity for thrombophilic disorders in neuro-COVID and hence headache patients should have a low threshold for investigations for cerebral venous sinus thrombosis (CVST) and secondary idiopathic intracranial hypertension (IIH) (55). IIH patients who are at high risk for impending visual loss need to be rapidly assessed. Based on a teleconsultation, patients who have a worsening of IIH could be brought to the hospital and in order to minimize hospital stay and improve compliance, a telemetric intracranial pressure monitoring or cerebrospinal fluid (CSF) shunting procedures may be considered. Patients with other chronic disabling headaches such as trigeminal autonomic cephalalgias and trigeminal neuralgia, who are scheduled for interventional procedures such as Gasserian ganglion blocks and multiple cranial nerve blocks, and neuromodulation treatment such as occipital nerve stimulation and DBS need to be reassessed on the treatment immediacy in COVID-19 period.

Necessary precautions must be taken by the healthcare workers and patients including the use of personal protective equipments (PPEs) and face masks to limit risks of possible COVID-19 transmission. Lumbar puncture and fundoscopy should be considered as aerosol-generating procedures and full PPE is recommended: double gloves, mask for the patient (for fundoscopy) and clinician. The clinician should also use an FFP3 mask (56). The removal and disposal of PPE are equally important.

## Stroke

Recent reports have indicated concerning trend of large vessel strokes in COVID-19 cases, especially among young COVID-19 patients who are asymptomatic or with mild symptoms (57). Stroke survivors at high risk of contracting the COVID-19 are the elderly, patients with the co-morbid disease such as obesity and those with swallowing difficulties. The most common comorbid diseases seen in COVID-19 patients are hypertension, diabetes, and coronary heart disease (58, 59). A study evaluating the clinical characteristics of 99 patients found cardiovascular and cerebrovascular diseases in 40% of patients as comorbid diseases (60). Patients are also relatively more susceptible to severe pneumonia in the case of contracting COVID-19. The medication regimen need not be changed in view of the present situation. In the event of limited access to outpatient services, patients should maintain regular compliance with medication and utilize telemedicine facilities to seek an appropriate expert opinion. Patients should follow the protocol of FAST symptom recognition and contact the emergency services. Triaging and rapid assessment will take into account the patient as well as the safety of the healthcare workers. An infection control and travel history screen would need to be completed by the paramedical team. Acute stroke protocols including “Protected Code Stroke” have been suggested for stroke patients in COVID-19 times (61). The current consortium has also developed a pathway targeting covering a broad spectrum of acute neurological emergencies, including acute ischemic stroke and transient ischemic attack (TIA), specifically for COVID-19 and takes into account considerations for infection risks and control toward minimizing exposure of healthcare workers during the entire continuum of acute stroke (62).

## Peripheral Neuropathy And Guillain-BarrÉ Syndrome

Neurological conditions affecting the PNS and muscle reported in COVID-19 include (63): peripheral motor neuropathy (64), Guillain-Barré Syndrome (GBS) (65–70), Miller Fisher syndrome (71), polyneuritis cranialis (71), acute myelitis (72, 73), and viral myopathy with rhabdomyolysis (74). Patients with peripheral neuropathy are not specifically at a greater risk of COVID-19 infection. However, precautions may be taken to prevent potential COVID-19 infection. A case of 69-year male from Northern Ireland has been reported presenting as peripheral motor neuropathy, with bilateral lower limb weakness, manifesting before the onset of the COVID-19 typical flu-like symptoms (64).

GBS is a post-viral autoimmune complication that was seen with both SARS and MERS. A case study of a 61-year-old lady from Wuhan who developed symptoms of GBS at the same time as COVID-19 showed the potential that GBS could be parainfectious, rather than just post-infectious, similarly seen with Zika virus (65). This report in isolation is inconclusive, but it indicates a need to consider potential neurological manifestations of COVID-19. A case series from Italy reported 5 cases of GBS with patients developing GBS symptoms in the mean of 5–10 days after onset of COVID-19 infection symptoms (66). It is not evident if having GBS prior to contracting COVID-19 will affect outcomes; however, if the patient is already ventilated or experiencing neuromuscular weakness this could confer additional risk (15). GBS is an acute and emergent presentation that can involve sensorimotor, bulbar or respiratory manifestation, dysautonomia, and nerve pain. In those that recover, it can be associated with life-long disabilities (75). It can also be fatal and has the potential to progress to Chronic Inflammatory Demyelinating Polyneuropathy (CIDP). The development of GBS associated with COVID-19 is hence a serious complication that could have permanent ramifications. It should be noted that GBS or CIDP patients who are being treated with chronic immunosuppressive drugs should be wary of increased infection risk and reduce exposure wherever possible (76). There should be tight surveillance of any accelerated increases in GBS diagnosis after the COVID-19 crisis rests due to the associated morbidity risks and disability.

## Neurovirological Manifestations

Studies investigating the neurovirological manifestations of COVID-19 are being conducted increasingly, as growing evidence demonstrates the ability of the virus to cross the blood-brain barrier and due to involvement of angiotensin-converting enzyme 2 (ACE2) receptor (77). The mechanism of entry into the CNS is not known, however, comparison to the SARS-COV and MERS-COV viruses display a common link across the neuroinvasive potential of coronaviruses (78). A retrospective study of 214 hospitalized patients with COVID-19 infection aimed at investigating the neurological manifestation of the disease, where 36.4% of the patients developed a CNS, PNS or skeletal muscle manifestations (5). Causes of suspicion include the virus being found in cerebrospinal fluid (CSF) samples and the symptom of anosmia. It is of particular concern that the virus could involve the brainstem.

A COVID-19-associated acute necrotizing haemorrhagic encephalopathy in a lady in her 50s has been reported (79). This is a rare form of encephalopathy that has been associated with other viruses (79). This complication is associated with a cytokine storm, and it is a consideration that cytokine storm syndrome could present in severe COVID-19 cases. Furthermore, cytokine storms associated with severe COVID-19 cases have been hypothesized to be related to secondary hemophagocytic lymphohistiocytosis (sHLH), a hyperinflammatory syndrome (80). It is proposed that immunosuppression may be necessary for such patients. This demonstrates the need to monitor inflammatory markers in COVID-19 positive patients. Furthermore, an investigation of associated neuroinflammation and COVID-19 would be beneficial, involving long-term follow-up and monitoring of early signs of neuroinflammation. This is important as neuroinflammation can translate to poor morbidity outcomes. It should hence also be a concern of health workers involved in the frontline clinical care of COVID-19 cases to monitor for any neurological changes, and neurologists could consider COVID-19 infection as a risk factor when encountering patients with new neurological manifestations in future.

## Autism and Pediatric Neurological Conditions

Most pediatric neurologists globally are offering telemedicine services for non-acute pediatric neurological disorders. It is quite challenging to have children at home, missing school, and doing school online while many parents are working from home. Autism Speaks has useful information and resources for children and adults affected with Autism spectrum disorders (81). Some of the information includes practical tips for parents, useful websites, school services, and information (81). Elective pediatric surgeries, those that are neither urgent or emergent, should be limited or rescheduled given the limited resources and anticipated surge in capacity to limit COVID-19 exposure to pediatric patients, their families and healthcare workers (82). Proactive approaches to identify pediatric neurological patients who might be at the risk of progressing from “semi-urgent” (non-symptomatic) to “urgent” (symptomatic) condition could be useful in preventing exposure due to emergency presentation.

## Alzheimer's Disease and Related Dementias

Patients with Alzheimer's disease and related dementias (ADRD) are at increased COVID-19 infection risk and its associated morbidity and mortality (83). They are less likely to adhere to public health restrictions and recommendations. Dementia patients have a higher prevalence of the cardiovascular disease, diabetes and pneumonia—which confers them an increased risk of severe illness after COVID-19 infection (83). The strain on healthcare services has also adversely impacted the diagnosis and clinical management of ADRD patients. ADRD patients living in a group and assisted living environments as well those in long-term care or nursing homes are especially vulnerable to an infection outbreak in their facilities (83, 84). Any outbreak can have disproportionately high attack rate and case fatality rate. Several authorities and governments have imposed strict restrictions on visits and access to these facilities. However, this has led to further social isolation and stress among residents (84). Close monitoring of patients via telemedicine is required. Identifying patients who are at “high-risk” of developing an acute event and continual assessment of the risk-benefit ratio of some medications are a priority (83).

## Mental Health Ramifications for Patients, Carers, and Providers

The psychological and psychiatric impact of COVID-19 on patients and carers is a matter of ongoing debate and concern (85). Given social distancing has been imposed by several governments; it will be relevant and also necessary to study the long-term negative consequences on both physical and mental health should this be prolonged. Patients with chronic conditions might be experiencing a sense of hopelessness and frustration during this crisis leading to non-compliance and potential relapse. Patients requiring carers will need to have backup plans in place to ensure continuity of care. For patients such as those with MS and MND who have experience managing infection risk, the extra measures such as social distancing and changes to care could add to anxiety about their health. As outlined by the European Association of Neurologists, patients with dementia are also affected by the closure of care facilities; they may become more agitated or anxious, there may be a breakdown in communication and live-in carers and family may experience deterioration in their own mental health (86). The Alzheimer's Disease International and international dementia expert panel has called for an urgent need of mental health and psychosocial support, in addition to physical protection from COVID-19 infection, for people living with dementia and their carers (84). Clinicians are experiencing an increasing number of calls with concerns about an irrational fear of contracting COVID-19 leading to worsening of a previous anxiety disorder, new-onset illness anxiety, and even engaging in repeated ritualistic behaviors such as excessive cleaning [obsessive-compulsive disorder (OCD)-like symptoms]. There are also reports on patients avoiding regular follow-ups. There is a worry as to what the delay in appointments and elective operations will mean for long-term patient outcomes.

The COVID-19 pandemic has had major impacts on the mental health of patients, carers and healthcare workers. The physical and emotional burnout among physicians is also a major long-term concern, and symptoms of burnout such as loss of empathy should be recognized (87). Another important consideration in the COVID-19 pandemic is the impact of adjustment disorder, acute stress disorder and post-traumatic stress disorder (PTSD) symptoms on clinicians, providers and those working in direct contact with patients. These individuals experience recurring exposure to the morbid effects on their patients as well as recurring exposure to the virus and thus awareness of threats to their own lives (88). It is imperative to actively investigate early PTSD symptoms, their trends and indications so that they could inform strategies for early and optimal treatment. The reorganization of medical staff is another point of stress for both staff and patients (85). As neurologists are being or soon-to-be repurposed, the ability of current patients to contact their physicians will be impacted and physicians will have to manage ongoing patients as well as increasing workload. It is harmful to patient care if there is ambiguity in the prioritization of patients. It is important to recognize the vulnerable nature of some of these patients during the pandemic and the necessity that they not be overlooked. A systematic review and meta-analysis on psychiatric and neuropsychiatric presentations associated with severe coronavirus infections (SARS, MERS, and COVID-19) observe, should SARS-CoV-2 follows a similar trajectory to that of SARS-CoV or MERS-CoV, a significant proportion of patients should recover without experiencing mental illness (89). However, the study highlighted the prevalence of delirium in acute stages in a significant proportion of COVID-19 patients (89). Long-term psychiatric manifestations, in COVID-19 infected patients, such as depression, PTSD, fatigue, anxiety, and rarer neuropsychiatric syndromes could be expected by clinicians (89). Telehealth or telepsychiatry services could provide mental health support during and beyond this pandemic (90).

## Recommendations and Discussions

Patients with chronic neurological diseases suffer from a disability, restricted mobility, and associated challenges that interfere with the independent quality of living. It is recommended that chronic neurological patients in general, and those with immunocompromised conditions in particular, must self-isolate if at high-risk and ensure that sufficient provision of medications are available for a prolonged time as the pandemic inflicted shut-down continues. Patients with chronic neurological conditions should be considered of higher risk of COVID-19 infection should they present to the hospital and appropriate personal protective safety equipment could be provided as per the clinical assessment. We propose a triage and management algorithm specific for patients with underlying chronic neurological diseases while following strict personal protective measures including PPEs for healthcare workers and patients as appropriate (Figure 1). Risk based triage of chronic neurological patients during the COVID-19 era is also proposed (Table 1). Patients with confirmed or suspected COVID-19 should be separated into COVID-19 Neuro and COVID-19 non-Neuro wards at the point of triage. Impact of COVID-19 on various chronic neurological conditions and corresponding additional recommendations from this consortium (along with existing guidelines) have been summarized in Table 2. We acknowledge that our algorithm may have limitations via a visa application in case of LMICs where facilities for neurological patients are limited or lacking. However, recommendations applicable to these settings have been provided for specific conditions such as epilepsy.

**Table 1:** Risk-based triage for non-acute neurological patients.

**Risk level**	**Characteristics**
Mild risk	· Person with no COVID-19 symptoms (fever, cough, sore throat, fatigue, shortness of breath, anosmia) · Person with mild COVID-19 symptoms, no shortness of breath, fever <38°C
Moderate risk	· Stable patient presenting with COVID-19 symptoms · Oxygen Saturation >92% in room air · Fever >38°C · Fatigue · Extra care should be taken with chronic neurological patients being treated with immunosuppressants or who have bulbar, respiratory or chewing issues Such patients should be placed within a neurology-only COVID-19 ward
Severe risk	· Patients presenting with both respiratory and systemic COVID-19 symptoms · Respiratory rate >30 brpm · Oxygen Saturation <92% in room air · Fever >38°C · Reduced alertness · Systolic blood pressure <90 mmHg · Diastolic BP <60 mmHg · Chronic neurological patients with severe muscle, bulbar and respiratory issues or who already require breathing assistance
Critical risk	· Patients in respiratory failure, hypotension, impaired consciousness or respiratory distress · Patients who are ventilated yet still deteriorated

Adapted from a–d:

a. Lewin E Push to include anosmia as a recognized COVID-19 symptom newsGP: racgp; 2020.

b. National COVID-19 clinical evidence taskforce Management of patients with moderate to severe COVID-19 disease: covid19evidence, 2020.

c. National COVID-19 clinical evidence task force Management of patients with severe to critical COVID-19 disease: covid19evidence, 2020.

d. All India Institute of Medical Sciences COVID-19 management protocol 2020.

**Table 2:** Summary of the impact of COVID-19 on chronic neurological conditions and recommendations for their management.

**Non-acute** **neurological condition**	**Impact of COVID-19** **infection/crisis**	**Recommendations/guidelines for** **management by professional** **bodies/health authorities**	**Recommendations by** **REPROGRAM consortium**
Multiple sclerosis	Reduced availability of services in the COVID-19 crisis Risk due to an immunocompromised state	Guidelines by some Multiple Sclerosis associations released on disease-modifying treatments (DMTs)	Telemedicine Consider long-term association with brain atrophy
Neuromuscular disorders (including Motor Neuron Disease)	At higher risk due to bulbar or respiratory weakness, may already require breathing assistance Higher risk of pneumonia Clinical trials halted Loss of trust in caregivers	Maintenance of breathing equipment Adequate supply of medication, essential items, and feeding tube supplies Strict social distancing, avoiding non-essential travel Ensure continuity of care through communication with caregivers	Use online order facilities Neurologists should be cognizant of interruptions to clinical trials
Epilepsy	Overrepresentation in LMIC Risk of fever-triggered seizures Possible mobility and cognitive disabilities	Do not cease antiepileptic medications Discussions with physicians about any current immunosuppressants Epilepsy Foundation guidelines	Rationing of non-acute neurological testing including cancellation of elective epilepsy monitoring could be explored Individual case-mix and case-by-case approach preferred Government should consider allowing pharmacists to refill epilepsy scripts during COVID-19
Parkinson's Disease and other movement disorders	Often elderly, vulnerable population Possible bulbar and respiratory issues Cognitive impairments could impact compliance Infections may lead to sudden motor and behavioral changes	Healthcare workers must have knowledge on PD and be prepared for delirium Masks and eye protection should be worn during Botox® procedures	Physicians should recognize that anosmia is already a PD symptom Nursing and care homes need to ensure PD patients stay quarantined Elective procedures (PEG, FUT, and DBS) should be postponed IPG battery replacements should still be performed Tele-exercise and tele-physiotherapy should be utilized
Migraine and severe headache	Require frequent outpatient consultations	Use Telemedicine	Minimize all non-emergent procedures If physical consultation is required, ensure telephone, and front-desk screening for COVID-19
Stroke	Residual impairments including dysphagia May be elderly with the comorbid disease Pneumonia risk	FAST protocol Protected-code stroke	Medication regime needs no change Use of telemedicine Triage, rapid assessment, and infection screening REPROGRAM Acute Stroke Pathway for broad spectrum of acute neurological emergencies including stroke and transient ischemic attack
Guillain-Barré Syndrome	Possible parainfectious profile Post-infection complication Potential for life-long disability and CIDP Maybe immunosuppressed	GBS and CIDP patients are only deemed at higher risk if on immunosuppressants	Tight surveillance of any accelerated increases in GBS diagnosis after the COVID-19 crisis rests
Neuro-virological manifestations	Involvement of ACE2 receptor, neurological involvement	Limited or non-specific guidelines	Monitor inflammatory markers and signs of neuroinflammation Monitor COVID-19 patients for any neurological change Neurologists could consider COVID-19 infection as a risk factor when encountering patients with new neurological manifestations in future
Autism and pediatric neurological conditions	Disruption to daily life and managing children at home	Autism Speaks guidelines	Limit elective pediatric surgeries Proactively identify patients at risk of progressing from semi-urgent to urgent

Prepared by the authors based on the evidence from the studies discussed in the paper.

*DMT, disease-modifying treatments; LMIC, low- and middle-income countries; CIDP, Chronic Inflammatory Demyelinating Polyneuropathy; COVID-19, severe acute respiratory syndrome coronavirus 2; ACE2, angiotensin-2 converting enzyme; PD, Parkinson's Disease*.

The role of population health and accurately coded data on COVID-19 patients is poignant as it could enable analysis and tracking of patients to help inform forward onward management of patients with similar dispositions and toward longitudinal follow-up to study the long-term impact on the physical and emotional health of COVID-19 patients with or without underlying neurological diseases. Technological solutions such as telemedicine consultation should be explored as a priority to minimize risk to patients and clinicians alike especially for patients with MS, PD, MG, ADRD, and ALS. Upper respiratory infections can be relatively severe and possibly fatal in immunocompromised, ALS, PD, and MG patients. It may be considered that neurologists collaborate with primary care physicians so that responsibilities could be shared especially at times when neurologists may be repurposed, and health systems are being reorganized for tending to frontline COVID-19 case triage and management. Comorbidities such as cardiovascular disease, diabetes, and obesity also warrant a targeted approach in COVID-19 (91) and this also applies to chronic neurological conditions who carry an increased risk of these comorbidities (92). Mental health implications of COVID-19, health system reorganization and self-isolation on patients with chronic neurological conditions warrant attention (9, 85, 89). Ongoing monitoring of mental health impact of COVID-19 on healthcare workers, patients and carers would help identify those at higher risk of mental health problems (90).

Institutions need to consider alternative ways to maintain continuity of care given the restrictions and limited availability of public transport amidst lockdowns, patients increasingly are at a disadvantage and sometimes unable to access regular or ongoing consultations. It is also relevant that chronic neurological patients would benefit from targeted education and outreach through their respective support networks or organizations around taking necessary anti-COVID-19 preventative measures such as hand hygiene, masks, etc. Patients must be encouraged to follow public health advice issued by their concerned authorities on hand hygiene, cough etiquette and should practice social distancing to minimize the COVID-19 infection and spread. Patients who are on immunosuppressant therapy, discussion with their physician and ongoing close monitoring in COVID-19 era should be undertaken.

Utilizing electronic patient records to understand the impact of underlying neurological conditions and comorbidities on the outcomes of patients who are COVID-19 positive will be fundamental. There also needs to be increased communication between neurologists and primary care physicians about compliance to and provision of medications, and physicians should be proactively cognizant of any relevant disruptions to a patient's clinical trials. Compliance to ongoing treatment is crucial and it is anticipated that this may be increasingly challenging for patients who are already under severe emotional distress and anxiety. Ensuring that such patients are able to seek timely and appropriate medical consultation will help ease their ongoing struggle and possibly limit non-compliance risks. The importance of email communication, outreach and touching base with patients should not be underestimated. Patients living in nursing homes or elderly care facilities should be encouraged to get pneumonia and influenza vaccinations. Carers and nursing home staff must take added precautions and measures to ensure that any suspected staff or visitors who may have COVID-19 symptoms should not be allowed in the facilities so that ongoing quarantine is maintained.

## Author Contributions

SBh devised the project, the main conceptual ideas, and proof outline. SBh and SBr wrote the first draft of the manuscript, and to investigate and supervised the findings of this work. SBh, SBr, SI-K, BM, VC, PT, JCh, RK, PP, DR, SG, AC, NS, HY, SMa, MO, EG, MT, NH, KM, JCo, and SMe discussed the results and recommendations, and contributed to the final manuscript. All authors contributed to the article and approved the submitted version.

## Conflict of Interest

The authors declare that the research was conducted in the absence of any commercial or financial relationships that could be construed as a potential conflict of interest. The Reviewer AK declared a shared affiliation with one of the author, SI-K to the handling editor at time of review.
